# Dual-Functioning Scaffolds for the Treatment of Spinal Cord Injury: Alginate Nanofibers Loaded with the Sigma 1 Receptor (S1R) Agonist RC-33 in Chitosan Films

**DOI:** 10.3390/md18010021

**Published:** 2019-12-26

**Authors:** Barbara Vigani, Silvia Rossi, Giuseppina Sandri, Maria Cristina Bonferoni, Marta Rui, Simona Collina, Francesca Fagiani, Cristina Lanni, Franca Ferrari

**Affiliations:** 1 Department of Drug Sciences, University of Pavia, Viale Taramelli, 12, 27100 Pavia, Italy; barbara.vigani@unipv.it (B.V.); giuseppina.sandri@unipv.it (G.S.); cbonferoni@unipv.it (M.C.B.); marta.rui@unipv.it (M.R.); simona.collina@unipv.it (S.C.); francesca.fagiani@iusspavia.it (F.F.); cristina.lanni@unipv.it (C.L.);; 2 Scuola Universitaria IUSS, Istituto Universitario di Studi Superiori, 27100 Pavia, Italy

**Keywords:** spinal cord injury, S1R agonist, chitosan, alginate, RC-33/ALG interaction product, electrospinning, film casting, mechanical properties, biodegradation, human neuroblastoma cells

## Abstract

The present work proposed a novel therapeutic platform with both neuroprotective and neuroregenerative potential to be used in the treatment of spinal cord injury (SCI). A dual-functioning scaffold for the delivery of the neuroprotective S1R agonist, RC-33, to be locally implanted at the site of SCI, was developed. RC-33-loaded fibers, containing alginate (ALG) and a mixture of two different grades of poly(ethylene oxide) (PEO), were prepared by electrospinning. After ionotropic cross-linking, fibers were incorporated in chitosan (CS) films to obtain a drug delivery system more flexible, easier to handle, and characterized by a controlled degradation rate. Dialysis equilibrium studies demonstrated that ALG was able to form an interaction product with the cationic RC-33 and to control RC-33 release in the physiological medium. Fibers loaded with RC-33 at the concentration corresponding to 10% of ALG maximum binding capacity were incorporated in films based on CS at two different molecular weights—low (CSL) and medium (CSM)—solubilized in acetic (AA) or glutamic (GA) acid. CSL- based scaffolds were subjected to a degradation test in order to investigate if the different CSL salification could affect the film behavior when in contact with media that mimic SCI environment. CSL AA exhibited a slower biodegradation and a good compatibility towards human neuroblastoma cell line.

## 1. Introduction

Spinal cord injury (SCI) is one of the most debilitating pathologies, that results in devastating neurological disabilities; it causes the loss of sensory and motor capabilities, in addition to other common complications, such as cardiac and respiratory dysfunctions, alterations in bladder and bowel control and loss of sexual function [1]. 

The World Health Organization states that, every year, between 250,000 and 500,000 people suffer a SCI worldwide [2]. The majority of SCIs is generally caused by a physical trauma that exerts compression on the spinal cord, triggering, in few minutes, a cascade of cellular, biochemical, and vascular responses; including inflammation, glutamate excitotoxicity, oxidative stress, and mitochondrial dysfunction. Such secondary injury mechanisms are involved in SCI progression, being responsible for an enlargement of the area of damage; they elicit oligodendrocyte apoptosis and, thus, axon demyelination, leading to the formation of a glial scar, which acts as both a physical and chemical barrier to any further attempts to promote neural repair and functional recovery [3]. The complex pathophysiology of SCI may explain the current lack of an effective therapeutic approach. Nowadays, the treatments can be classified as neuroprotective or neuroregenerative ones [4,5]. 

Neuroprotective drug-based therapies are intended to avoid or prevent further progression of the secondary injury. Recently, it was demonstrated that some of the above-mentioned secondary mechanisms of damage may be related to a dysfunction of Sigma 1 Receptor (S1R), suggesting that this receptor could be a valuable target for developing new drugs to treat SCI [6]. In particular, accumulating evidence highlighted the potential role of S1R agonists in exerting neuroprotective and/or restorative effects, by reducing excitotoxicity and oxidative damage [7,8,9,10,11]. Along the years, Rossi et al. designed and synthetized a S1R modulator compound library, belonging to the common arylalkyl(alkenyl) aminic scaffold, which led the identification of 1-[3-(1,1′-biphen)-4-yl]butylpiperidine (henceforth RC-33), as a molecule endowed with the most promising pharmacological profile and able in promoting nerve growth factor(NGF)-induced neurite outgrowth in PC-12 cells [12,13,14,15]. In the light of these properties, RC-33 was selected as a neuroprotective agent to further investigate the role of S1R agonists in treating SCI.

On the counter part, neuroregenerative approaches are directed to recover the lost or impaired functionality of the spinal cord by designing and manufacturing biomaterial-based scaffolds with physico-chemical and mechanical properties tailored to repair the broken neural circuitry of spinal cord [4]. Among such biomaterials, chitosan (CS) and alginate (ALG)—which are the two most commonly used marine derived polysaccharides in both drug delivery and tissue engineering—have recently gained interest in the development of scaffolds to treat SCI.

CS is a linear polysaccharide derived from the partial deacetylation of chitin that represents the main component of mollusk and crustacean exoskeleton; it is characterized by randomly distributed glucosamine and *N*-acetylglucosamine units linked through β(1–4) glycosidic bonds [16]. In the literature, CS has been largely recognized as a multifunctional polymer thanks to its biocompatibility; tunable degradation kinetics; and its mucoadhesive, antimicrobial, and hemostatic potential [17,18,19,20]. Moreover, CS possesses antioxidant and radical scavenger properties [17,21], which could be profitably employed to counteract the increase in the production of ROS that contributes to the spinal cord motor neuron death upon spinal cord injuries [22,23]. 

In the context of SCI, CS tubes or channels, empty [24], filled with type I collagen [25], l-poly-lysine [26], peripheral nerve graft or neural stem/progenitor cells [27,28], have been directly implanted in the injured spinal cord of rat models, proving to be able of guiding and supporting axon regrowth and, thus, of bridging the gap of the lesion. Moreover, CS exhibits in vitro neuroprotective effects on several neuronal cell lines, by exerting anti-inflammatory activities and protecting from apoptotic mechanisms [29]. More recent studies focused on the development of CS porous matrices, solutions, and hydrogels, have revealed that CS scaffolds—when implanted at the site of SCI in the sub-acute stage—provide a protective and neurotrophic microenvironment that is functional both to the survival of damaged endogenous neurons and to the differentiation of transplanted stem cells into neuronal ones [28,30,31,32]. 

ALG is an anionic polysaccharide, generally extracted from brown algae (*Phaeophyceae* species). It is a linear copolymer containing blocks of α-l-guluronic acid (G) and β-d-mannuronic acid residues linked by a β-1,4-glycosidic bond and it can be cross-linked by complexation of its carboxylic groups with divalent cations, such as Ca^2+^ ones [33]. Due to its biocompatibility and versatility, ALG is commonly used as scaffolding material in drug delivery, tissue engineering applications and cell encapsulation [34]. In the last decade, ALG-based anisotropic capillary hydrogels have been designed in order to ensure a neuroregenerative effect following SCI, providing an effective physical guiding structure able to support the axonal longitudinally oriented regrowth both in vitro and in vivo [35,36,37,38]. 

Nowadays, the development of therapeutic platforms, with both neuroprotective and neuroregenerative potential, able to guarantee a controlled drug delivery, as well as the neurite outgrowth at the site of injury, represents a current challenge in the biomedical and pharmaceutical fields. In the context of SCI, polymer-based fibers, produced by the electrospinning technique, are considered both as attractive bridging materials, filling the gap in the spinal cord [39,40,41,42], and as effective drug delivery systems, thanks to their high area/volume ratio that maximize the cellular uptake of the drug released [40,43]. 

Given these premises, the present work deals with the formulation development of a potential dual-functioning scaffold for the delivery of the neuroprotective S1R agonist, RC-33 [12,13], to be locally implanted at the site of SCI. 

In particular, RC-33-loaded fibers, containing ALG and a mixture of two different grades of poly(ethylene oxide) (PEO), having high (h-PEO) and low (l-PEO) molecular weights (MWs), were prepared by the electrospinning technique. ALG was selected as the main component of fiber matrix since it is an anionic polysaccharide able to form an electrolyte complex or interaction product (IP) with the cationic RC-33, whereas the combination of two different PEO MWs allowed the production of electrospun nanofibers, characterized by optimal mechanical properties [44]. The novelty of the paper resides in the development of a nanofiber scaffold based on the combination of the anionic alginate with the cationic RC-33. At the best of our knowledge, to date the formation of an RC-33/ALG IP and the possibility to load it in electrospun nanofibers have not been studied. The stoichiometry of RC-33/ALG IP and its drug release properties have been investigated by dialysis equilibrium studies in distilled water and in physiological medium, respectively.

In an attempt to produce a drug delivery system that is flexible, easier to handle, and characterized by a controlled degradation rate and by peculiar surface properties, RC-33 loaded fibers were cross-linked with CaCl_2_ aqueous solutions [45] and, then, incorporated in CS films. Chitosan was chosen in order to improve scaffold regenerative potential. The developed scaffolds were characterized in terms of morphological properties, mechanical resistance, phosphate buffer uptake, and wettability. Their degradation was also evaluated after soaking in solutions mimicking the SCI environment rich in agents responsible for the oxidative stress or enzymes over-expressed at the site of injury. Finally, preliminary in vitro cell studies were performed to evaluate the cytotoxicity of such scaffolds on SH-SY5Y human neuroblastoma cell line.

## 2. Results and Discussion

### 2.1. Synthesis of RC-33 and Assessment of ALG/RC-33 Interaction

**Figure 1:**
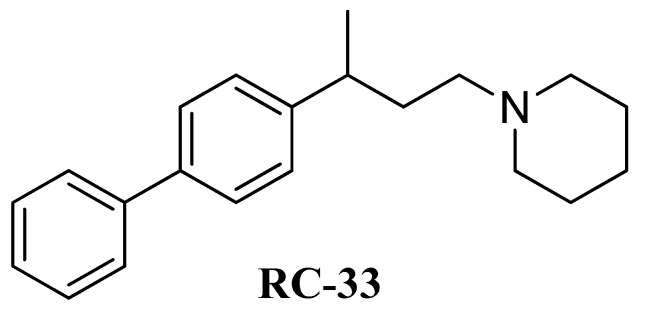
Chemical structure of RC-33.

As for the synthesis of RC-33, the same approach reported in a previous work [13] was used to obtain the desired product in a 10-grams scale, a sufficient amount to perform the subsequent studies (see Supplementary Materials). In Figure 1, the chemical structure of RC-33 is reported. 

**Figure 2:**
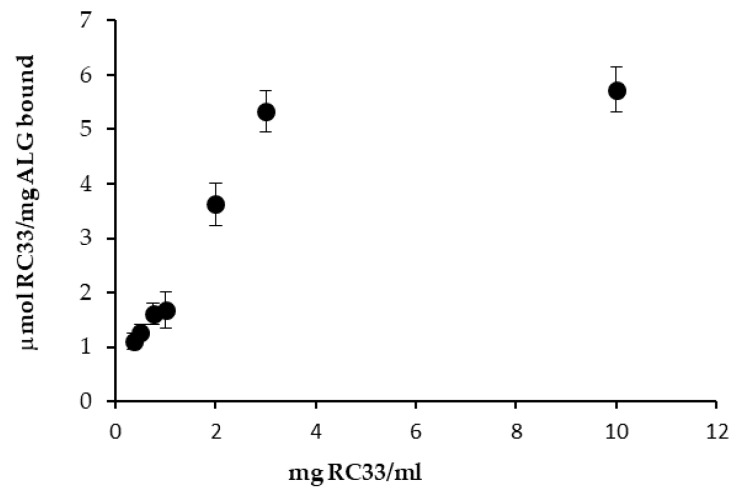
RC-33 μmoles bond to 1 mg of ALG vs. RC-33 concentration profile (means values ± s.d.; *n* = 3).

Figure 2 shows the results obtained from dialysis equilibria. In particular, values of RC-33 μmoles bond to 1 mg of ALG are reported as a function of RC-33 concentration (mg/mL). ALG concentration was fixed and equal to 1% *w*/*v*. For all the drug concentrations considered, a precipitate forms in the dialysis bag, indicating the formation of an interaction product (IP) between RC-33 and ALG.

It can be observed that for drug concentrations higher than 3 mg/mL, the drug amount bond to the polymer remains virtually unchanged. This indicates that the maximum binding capability of the polymer is reached. Such capability corresponds to 1.76 mg of drug for 1 mg of polymer. 

**Figure 3:**
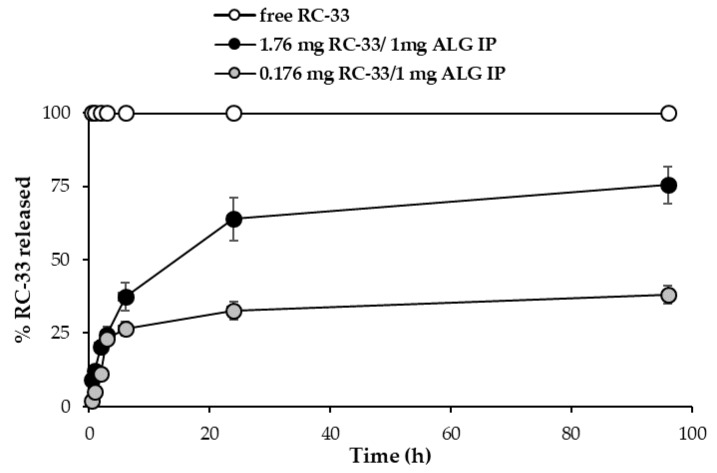
RC-33 release profiles from the suspension obtained by adding 176 mg of RC-33 to 10 mL of 1% (*w*/*w*) ALG (1.76 mg RC-33/1 mg ALG IP) and from the suspension obtained by adding 17.6 mg of RC-33 to 10 mL of 1% (*w*/*w*) ALG (0.176 mg RC-33/1 mg ALG IP). The dissolution profile of the free drug is also reported (means values ± s.d.; *n* = 3).

Figure 3 reports the RC-33 release profiles from (i) the suspension obtained by adding 176 mg of RC-33 to 10 mL of 1% (*w*/*w*) ALG corresponding to the ALG maximum binding capacity (1.76 mg RC-33/1 mg ALG IP) and (ii) the suspension obtained by adding 17.6 mg of RC-33 to 10 mL of 1% (*w*/*w*) ALG corresponding to 10% of the ALG maximum binding capacity (0.176 mg RC-33/1 mg ALG IP). The dissolution profile of the free drug is also reported. 

The results obtained points out the capability of RC-33/ALG IP of guaranteeing a prolonged release of RC-33, probably due to an exchange between the RC-33 and the sodium ions of the physiological medium. The lower profile observed for the 0.176 mg RC-33/1 mg ALG IP (10% of ALG maximum binding capacity) is probably due to the slowing effect of the ALG chains free of drug that form a viscous environment through which RC-33 diffuses.

### 2.2. Preparation of Nanofibers Loaded with RC-33 by Electrospinning

It is generally recognized that polymer solution properties, such as rheology, surface tension and conductivity affect the morphology of the fibers obtained by electrospinning. 

In particular, a high viscosity at high shear rates should enable the formation of bead-free fibers. In fact, the disruption of the polymeric jet with the consequent bead formation into the fibers could occur in presence of polymer solutions characterized by low viscosity [46,47]. During the electrospinning process, the surface tension of the polymer solution influences the formation of Taylor’s cone; in general, a decrease of surface tension, due to the addition of surfactants such as poloxamer, enables fiber formation at low electric fields [48,49]. As for conductivity, a polymer solution lacking in charges does not produce fibers by electrospinning; an increase in electrical charge generally improves the elongation capability of the solution, favoring the formation of smaller diameter smooth fibers [48,50]. In a previous work of ours, a DoE approach was used to draw up guidelines for the preparation of mixtures of ALG with non-ionic polymers able to produce homogeneous nanofibers by electrospinning [45]. In particular, optimal values for viscosity at high shear rates, surface tension and conductivity were identified. In order to investigate whether the presence of RC-33 affects such properties and, in turn, the formation of electrospun fibers, an ALG/PEO mixture previously developed [44], characterized by optimized properties and able to form nanofibers, was loaded with two different RC-33 concentrations corresponding to 10% (10% LOADED) and 30% (30% LOADED) of ALG maximum binding capacity. In fact, it is necessary that not all ALG negative charges interact with the cationic drug since such charges are responsible for solution conductivity (functional to solution electrospinnability as described above) and for the possibility of fiber ionotropic cross-linking. Polymer-drug mixtures were characterized for viscosity, surface tension, and conductivity and the results obtained were compared with those of the mixture free of RC-33 (BLANK). 

**Figure 4:**
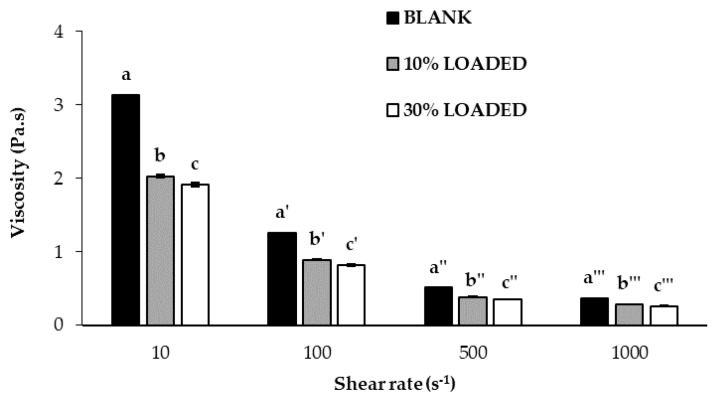
Viscosity values, measured at 10, 100, 500, and 1000 s^−1^ and at 33 °C (temperature employed for the electrospinning process), of the polymer solutions under investigation (mean values ± s.d.; *n* = 3). ANOVA one-way; Multiple Range Test (*p* ≤ 0.05): a vs. b, c; b vs. c; a’ vs. b’, c’; b’ vs. c’; a″ vs. b″, c″; b″ vs. c″; a‴ vs. b‴, c‴; b‴ vs. c‴.

In Figure 4, viscosity values of BLANK, 10% LOADED and 30% LOADED have been reported. Since they are characterized by decreasing viscosity values on increasing shear rates, all samples show a pseudoplastic behavior that is typical of polymer solutions. The presence of RC-33 is responsible for a viscosity reduction due to the formation of a RC-33/polymer IP [51,52]. In particular, the presence of RC-33 at the highest concentration (30% LOADED) produces a significant (*p* < 0.05) decrease of the viscosity (at all the shear rates considered) with respect to the value observed for 10% LOADED solution. Since a reduction of viscosity is recognized as an index of the formation of an IP [51,52], the results obtained confirm that the increase of RC-33 concentration is responsible for a greater RC-33/ALG interaction.

The presence of RC-33 at the two different concentrations does not affect the surface tension and the conductivity of the polymer solutions that result equal to 35–40 mN/mm and 2000–2100 μS/cm, respectively. Moreover, a variation in process parameters, such as applied voltage (from 20 to 22 kV) and spinneret–collector distance (from 20 to 22 cm), was needed in presence of RC-33 to obtain a stable Taylor’s cone during the electrospinning process. 

**Figure 5:**
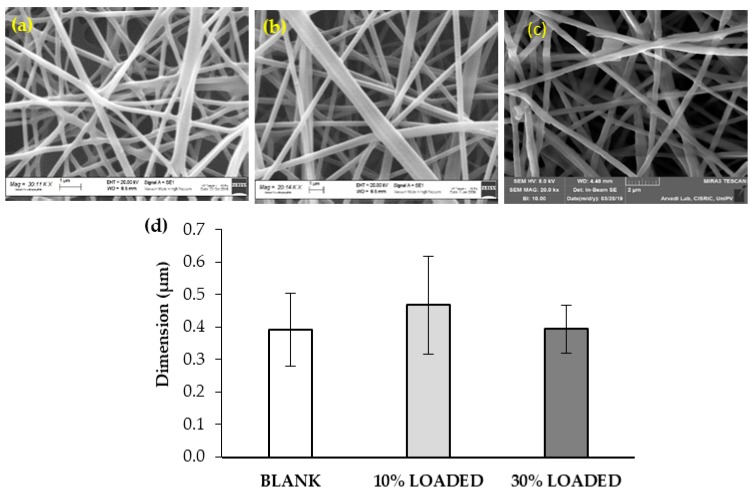
SEM images of (**a**) BLANK, (**b**) 10% LOADED, and (**c**) 30% LOADED nanofibers and (**d**) their dimensions (mean values ± s.d.; *n* = 30).

In Figure 5, SEM images and dimensions of the RC-33 loaded nanofibers have been compared to those of nanofibers free of RC-33. 

All polymer solutions under investigation produce homogeneous fibers with a diameter in the range 400–500 nm. The presence of some surface irregularities can be observed in the nanofibers loaded with the highest RC-33 concentration (30% LOADED). This can be due to an incomplete incorporation of RC-33/ALG IP into fibers. The presence of RC-33 does not produce any significant variation in nanofiber dimension. The fiber loading capacity was equal to 72 ± 3.3% and 65 ± 3.2% for 10% LOADED and 30% LOADED nanofibers, respectively (mean values ± s.d.; *n* = 3).

**Figure 6:**
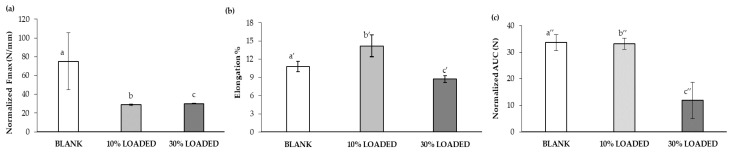
Mechanical properties of BLANK, 10% LOADED and 30% LOADED nanofibers: (**a**) normalized maximum deformation force (Fmax), (**b**) elongation %, and (**c**) normalized deformation work (AUC) (mean values ± s.d.; *n* = 3). ANOVA one-way; Multiple Range Test (*p* ≤ 0.05): (**a**) a vs. b, c; (**b**) a’ vs. b’, c’; b’ vs. c’; (**c**) a″ vs. c″; b″ vs. c″.

In Figure 6, the mechanical properties of the loaded nanofibers have been compared to those of the nanofibers free of RC-33. The presence of RC-33 is responsible for a drastic reduction of the normalized maximum deformation force (Fmax), thus indicating that the RC-33/polymer IP produces a weakening of the fibrillar network (Figure 6a). As for the normalized deformation work, calculated as the area under force vs. displacement curve (AUC), a reduction of this parameter is observed only for 30% LOADED nanofibers, while 10% LOADED nanofibers are characterized by an unchanged value with respect to BLANK nanofibers (Figure 6c); this result is associated with a greater percentage of elongation (elongation%) of 10% LOADED fiber to indicate a greater flexibility/plasticity of this sample (Figure 6b). On the basis of the results so far obtained, the subsequent experiments have been performed with the 10% LOADED formulation due to the better surface morphology free of irregularities and plasticity/flexibility properties.

**Figure 7:**
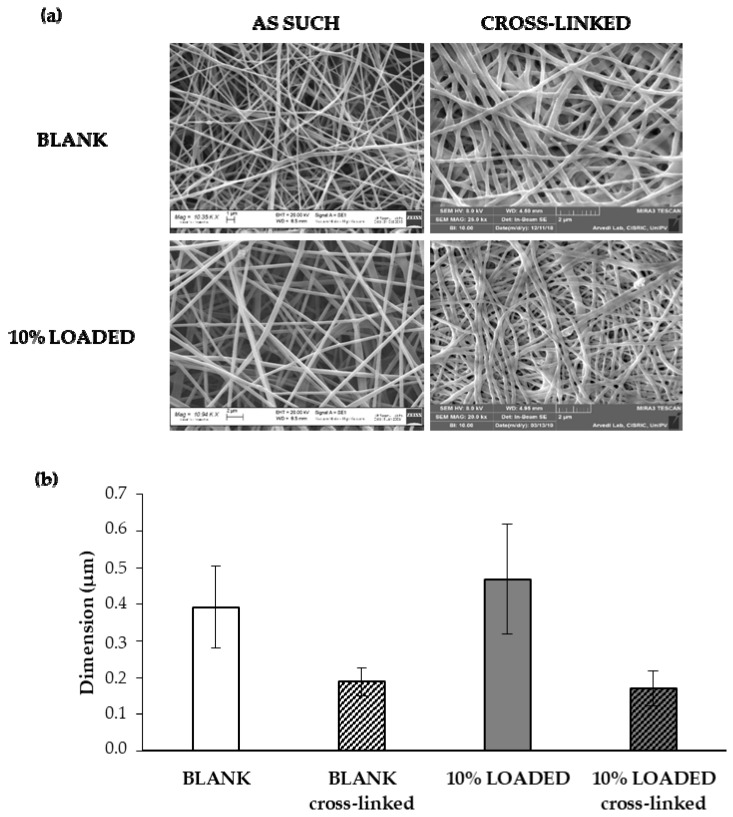
(**a**) SEM images and (**b**) dimensions (mean values ± s.d.; *n* = 30) of BLANK and 10% LOADED nanofibers, as such and upon cross-linking.

In order to make nanofibers insoluble in the physiological medium, BLANK and 10% LOADED nanofibers have been subjected to a cross-linking process with CaCl_2_. Figure 7 reports SEM images (**a**) and dimensions (**b**) of 10% LOADED nanofibers in comparison with BLANK ones, as such and upon cross-linking. The cross-linking process is responsible for a drastic reduction of fiber dimensions, attributable to the dissolution and removal of PEO and P407. These results are in line with our previous data [45]. Fiber size reduction leads to a contraction of fiber network with a consequent pore size decrease: 2.94 ± 0.200 μm and 1.45 ± 0.105 μm are the mean pore sizes of BLANK before and after cross-linking, respectively. The presence of RC-33 does not produce any modification in fiber and pore size. Upon cross-linking, the RC-33 content remains unchanged.

### 2.3. Preparation and Characterization of CS Films

Films based on two different CS MWs (low, CSL; and medium, CSM), solubilized in acetic acid (AA) or glutamic acid (GA), were prepared by casting. In the Supplementary Materials, the rheological properties (viscosity and viscoelasticity) of the CS solutions employed for film preparation have been reported. 

**Figure 8:**
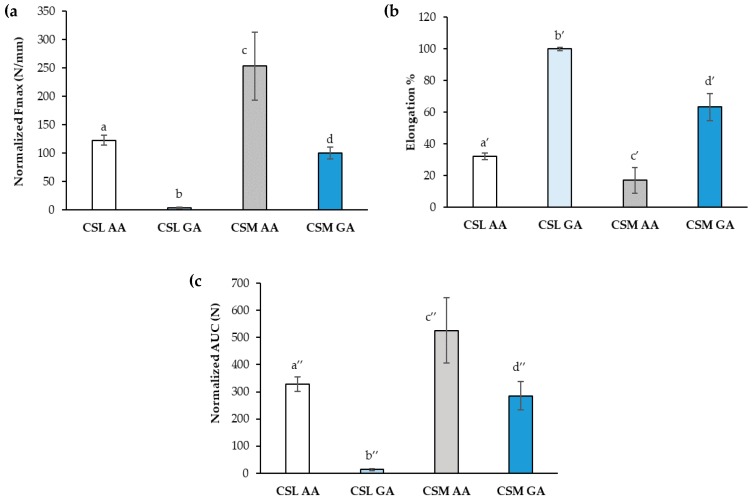
Mechanical properties of the films based on CSL AA, CSL GA, CSM AA, and CSM GA: (**a**) normalized maximum deformation force (Fmax), (**b**) percentage of elongation (elongation%), and (**c**) normalized deformation work (AUC) (mean values ± s.d.; *n* = 3). ANOVA one-way; Multiple Range Test (*p* ≤ 0.05): (**a**) a vs. b-d; b vs. c, d; c vs. d; (**b**) a’ vs. b’, c;’ b’ vs. c’; (**c**) a″ vs. b″-d″; b″ vs. c″, d″; c″ vs. d″.

Film mechanical properties expressed by normalized maximum deformation force (Fmax), percentage of elongation (elongation%) and normalized deformation work (AUC), have been evaluated (Figure 8). Considering the same CS MW, CS salification with AA produces films characterized by a higher mechanical resistance. In fact, films based on CSL AA and CSM AA show higher values of normalized Fmax and normalized AUC with respect to CSL GA and CSM GA, respectively (Figure 8a,c). As expected, the comparison of the data obtained for the films based on the two CS grades, at parity of acid, points out an increase in film mechanical resistance on increasing MW. As for elongation, at parity of acid, the films based on CSL are characterized by the greatest flexibility/plasticity, showing the highest values of elongation% (Figure 8b). Since film plasticity that is the capability of the film to deform itself without breaking [53] is functional to an easy administration, avoiding the risk of film breaking during its handling, CSL AA and CSL GA have been chosen for the prosecution of the work. 

**Figure 9:**
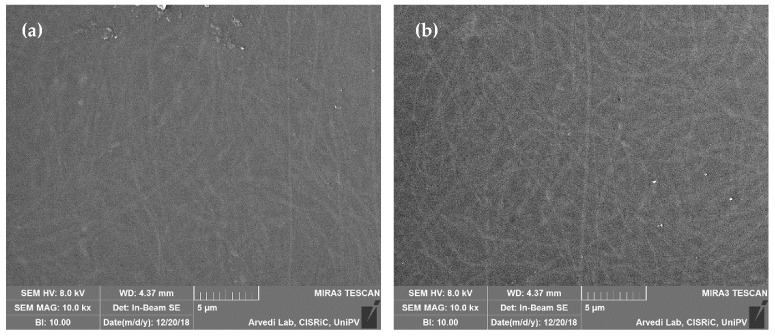
SEM images of dual-functioning scaffolds: (**a**) CSL AA and (**b**) CSL GA films loaded with 10% LOADED nanofibers.

Figure 9 shows SEM images of the dual-functioning scaffolds that consist of CSL AA and CSL GA films loaded with 10% LOADED nanofibers. The nanofiber network, immersed in CS matrix, is clearly visible and produces topographical cues of nanometric magnitude that should enhance and guide axon outgrowth [40].

**Figure 10:**
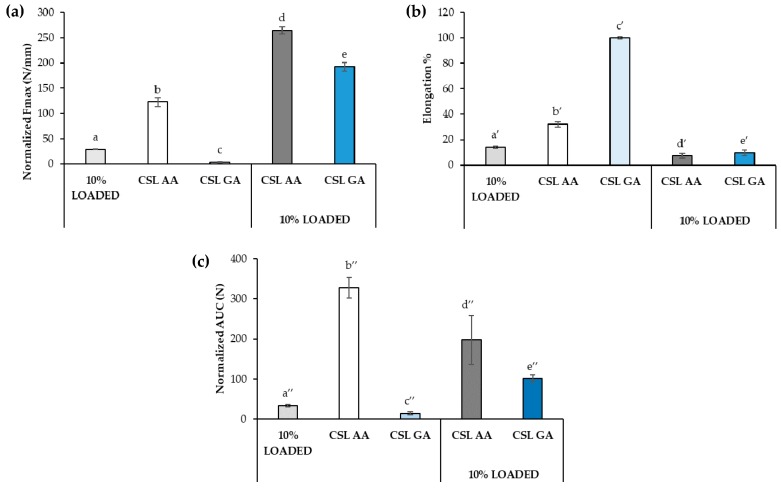
Mechanical properties of the films based on CSL AA and CSL GA in presence and in absence of nanofibers: (**a**) normalized maximum deformation force (Fmax), (**b**) percentage of elongation (elongation%), and (**c**) normalized deformation work (AUC) (mean values ± s.d.; *n* = 3). ANOVA one-way; Multiple Range Test (*p* ≤ 0.05): (**a**) a vs. b-e; b vs. c-e; c vs. d, e; d vs. e; (**b**) a’ vs. b’, c’; b’ vs. c’; (**c**) a″ vs. b″-e″; b″ vs. c″-e″; c″ vs. d″, e″; d″ vs. e″.

Figure 10 reports the mechanical properties of the dual-functioning scaffolds (CSL AA + 10% LOADED and CSL GA + 10% LOADED) in comparison with 10% LOADED nanofibers, CSL AA and CSL GA films. As for the normalized Fmax (Figure 10a), the presence of the nanofibers produces a significant increase in such parameter, indicating the occurrence of a strengthening of the structure, more marked for the scaffold composed of CSL GA film that was characterized, in absence of fibers, by the lowest mechanical resistance. Such strengthening produces a reduction in the percentage of elongation that is estimated for both scaffolds as around 10%, close to that observed to nanofibers alone (Figure 10b). As for normalized deformation work (AUC), a different behavior can be observed depending on film composition. In particular, the presence of the nanofibers produces a significant reduction of the normalized AUC of CSL AA scaffold; on the contrary, an increase in such a parameter is observed upon nanofiber incorporation into CSL GA film (Figure 10c). For such film, the marked increase in normalized Fmax (by almost two orders of magnitude) counterbalances the reduction of the elongation. Both dual-functioning scaffolds are characterized by AUC values significantly higher than that of nanofibers alone, indicating that the incorporation in film confers higher mechanical resistance to nanofibers.

**Figure 11:**
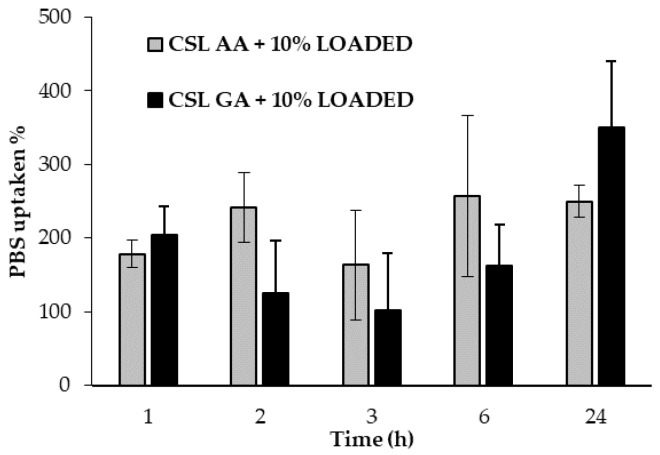
Hydration properties of dual-functioning scaffolds: % PBS uptaken values as a function of time (mean values ± s.d.; *n* = 3).

Figure 11 reports the hydration properties, expressed as % phosphate buffer solution (PBS) uptaken as a function of time, of both dual-functioning scaffolds. No significant differences have been observed between the two samples, which are able to absorb more than 200% of their weight.

**Figure 12:**
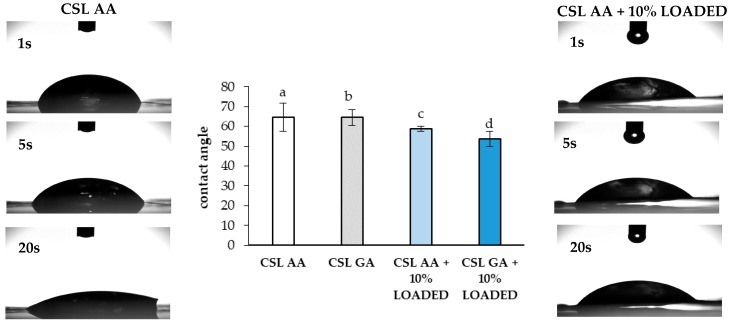
Contact angle values observed after 20 s for the CS-based films with and without 10% LOADED nanofibers (mean values ± s.d.; *n* = 3). As an example, images of the drop at different times (1, 5, and 20 s) are reported for CSL AA film in presence and in absence of the nanofibers. ANOVA one-way; Multiple Range Test (*p* ≤ 0.05): a vs. c; b vs. d.

In addition, the values of contact angle observed for the films based on CSL AA and CSL GA with and without 10% LOADED nanofibers have been measured (Figure 12). The presence of the nanofiber network produces a slight decrease in contact angle, probably due to a change in surface roughness [54]. All the samples are characterized by contact angles lower than 70°, indicating good wetting properties. 

**Figure 13:**
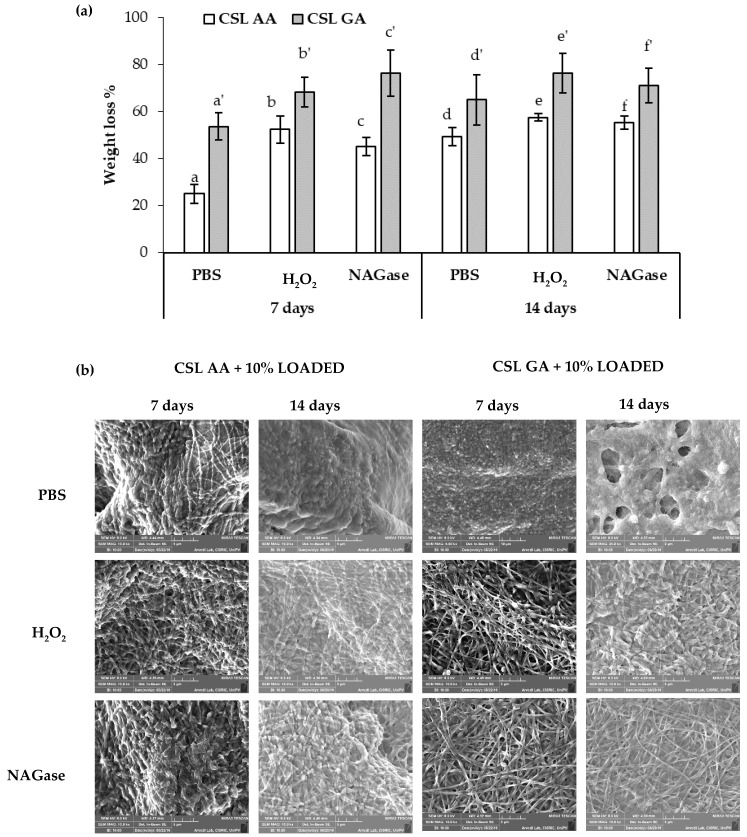
Biodegradation test: (**a**) weight loss % values (mean values ± s.d.; *n* = 3–5) and (**b**) SEM images of dual-functioning scaffolds (CSL AA and CSL GA films containing 10% LOADED nanofibers) immersed at 37 °C for 7 and 14 days in PBS, H_2_O_2_ 1.25 mM in PBS and NAGase 5 U/L in PBS. ANOVA one-way; multiple range yest (*p* ≤ 0.05): (**a**) a vs. d-e, a’-d’; b vs. b’, e’, f’; c vs. b’, c’, e’, f’; d vs. b’, c’, e’, f’; e vs. e’, c’; f vs. c’, e’, f’; a’ vs. c’, e’ f’, d’ vs. e’, f’.

In order to investigate whether the different salification of CS could affect the biodegradation of the formulation, both scaffolds, consisting of CSL AA and CSL GA films loaded with 10% LOADED nanofibers, have been placed in contact for 7 and 14 days with (i) PBS, (ii) H_2_O_2_ 1.25 mM in PBS, and (iii) β-*N* acetylglusosaminidase (NAGase) 5 U/L in PBS. Such conditions have been chosen to mimic the SCI environment. In particular, an increase in the production of ROS is recognized as an early and likely causal event contributing to the spinal cord motor neuron death [22,23]. Excessive production of ROS is responsible for a damage of the lysosomal membrane with the release of enzymes that, in turn, lead to cell death. Neurotrauma could cause lysosomal membrane damage, thus resulting in pathological autophagosome accumulation in the spinal cord. The autophagy–lysosome pathway may be a part of secondary injury processes of SCI [55]. Abraham et al. observed in an animal model an increase in NAGase upon contusion injury of spinal cord [56]. Figure 13 reports weight loss % values (a) and SEM images (b) of CSL AA and CSL GA based scaffolds after soaking in PBS, H_2_O_2_ 1.25 mM in PBS and in β-*N* acetylglusosaminidase (NAGase) 5 U/L in PBS for 7 and 14 days at 37 °C. After 7 days of contact, for both scaffolds, the presence of H_2_O_2_ and NAGase produces a significant increase in film biodegradation. After 14 days of contact, significant differences in weight loss % values have been observed among the different conditions (PBS vs. H_2_O_2_ or NAGase) only for CSL GA. For both the scaffolds and independently of the contact time, no significant differences have been observed between the data obtained in presence of H_2_O_2_ and NAGase. At parity of conditions, CSL AA scaffold is characterized by significantly lower weight loss % values with respect to CSL GA one, indicating a lower biodegradation. For this reason, they could support for a more prolonged period of time the outgrowth of neural cells [57].

**Figure 14:**
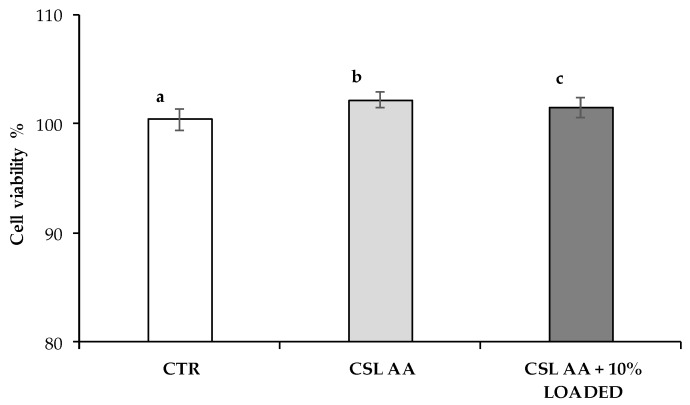
Cell viability% values observed for CSL AA film as such and containing 10% LOADED nanofibers; culture medium was used ad reference (CTR) (mean values ± s.d.; *n* = 3–6). ANOVA one-way; multiple range test (*p* ≤ 0.05): a vs. b, c.

CSL AA based scaffolds have been investigated for their cytotoxicity on SH-SY5Y human neuroblastoma cells. LDH released in the cell culture medium after 24 h of contact with the samples (CLS AA films with and without 10% LOADED) was measured and expressed as cell viability% with respect to the control (CTR, culture medium not in contact with the samples) (Figure 14). LDH enzyme, normally present in cell cytoplasm, is poured into the culture medium following damage to cell wall. Both the two samples show significantly higher cell viability% when compared to the control, it suggests good compatibility on SH-SY5Y human neuroblastoma cells. 

## 3. Materials and Methods 

### 3.1. Materials

Alginic acid sodium salt, from brown algae (ALG, medium viscosity, Brookfield viscosity ≥ 2000 cP for 2% wt. solution in water at 25 °C, G/M ratio equal to 70/30; Sigma-Aldrich, Milan, Italy), Kolliphor P407 poloxamer (P407; BASF SE, Ludwigshafen, Germany), poly(ethylene oxide) of high molecular weight (h-PEO, *MW* = 4000 kDa, Colorcon, Dartford, United Kingdom) and poly(ethylene oxide) of low molecular weight (l-PEO, *MW* = 600 kDa, Sigma Aldrich, Milan, Italy) were used for the preparation of ALG/PEO solutions. Anhydrous calcium chloride (CaCl_2_) and absolute ethanol (EtOH) were purchased from Carlo Erba Reagents S.r.l. (Milan, Italy) and used for ALG/PEO fiber cross-linking.

Acetic acid glacial (Carlo Erba Reagents S.r.l., Milan, Italy), chitosan medium *MW* (CSM, degree of deacetylation 75–85%, *MW* 190,000–310,000 Da, Brookfield viscosity = 200–800 cP for 1% wt. solution in 1% acetic acid at 25 °C; Sigma-Aldrich, Milan, Italy), ChitoClear (chitosan low *MW*, CSL; degree of deacetylation 98%; Primex, Siglufjordur, Iceland), glycerol 30°Bé (Carlo Erba Reagents S.r.l., Milan, Italy) and l-glutamic acid (Sigma Aldrich, Milan, Italy) were used for the preparation of CS films. 

Reagents and solvents for RC-33 synthesis were obtained from Merck (Italy). Solvents were purified according to the guidelines in Purification of Laboratory Chemicals. Solvents used for chromatography were HPLC grade and supplied by Carlo Erba (Milan, Italy).

### 3.2. RC-33 HCl Synthesis 

RC-33 was synthesized as described in [13]. Herein, the characterization of RC-33 HCl was reported. *Rf* = 0.40 (TLC: 8/2 AcOEt/MeOH, *v*/*v*). mp 2088–2094 °C; IR (cm^−1^) 3053, 2948, 2605, 2478, 1487, 1450, 1441, 1401, 1065, 1008, 959, 836, 766, 735; ^1^H NMR (D_2_O) δ (ppm): 12.10 (brs, 1H, H salt) 7.61 (m, 4H, Ar); 7.45 (m, 2H, Ar); 7.32 (m, 3H, Ar); 3.35 (m, 2H, ArCHCH_2_CH_2_); 2.97 (m, 1H, ArCH); 2.73 (m, 4H, Pip); 1.96 (m, 2H, ArCHCH_2_); 1.78 (m, 2H, Pip); 1.69 (m, 1H, Pip), 1,32 (m, 2H, Pip); 1.24 (m, 1H, Pip); 1.22 (d, 3H, ArCHCH_3_).

Melting points were measured on SMP3 Stuart Scientific apparatus and are uncorrected. For FT-IR analysis a Spectrum One Perkin Elmer spectrophotometer equipped with a MIRacle™ ATR device was used.

### 3.3. Binding and Drug Release Study by Dialysis Equilibrium 

Dialysis equilibrium studies were performed to investigate the polymer/drug interaction [58], in particular to quantify the amount of RC-33 bound to 1 mg of ALG. Briefly, 1% *w*/*v* ALG solution was enclosed in a dialysis bag (seamless cellulose tubing, cut-off > 12,000 Da, Sigma Aldrich, Milan, Italy), previously boiled for 15 min in distilled water and carefully washed, and, then, dialyzed towards a RC-33 HCl solution under mild stirring at room temperature until equilibrium was reached (24 h). Both ALG and RC-33 solutions were prepared in Milli-Q water and different RC-33 HCl concentrations were considered: 0.357, 0.5, 0.75, 1, 2, 3, and 10 mg/mL. 

The dialysis bag did not allow the polymer to get out, but allowed the drug to diffuse into and, eventually, to interact with the polymer. Drug concentration outside the dialysis bags was quantified by a HPLC method. HPLC-PDA-UV/Vis analyses were conducted on a Jasco (Cremella, Lecco, Italy) HPLC system, consisting of PU-1580 pump, 851-AS auto-sampler, and MD-1510 Photo Diode Array (PDA) detector. Experimental data were acquired and processed by Jasco Borwin PDA and Borwin Chromatograph Software. Analyses were run on a Symmetry C18 (5 μm, 3.9 × 150 mm) column, at room temperature, with gradient elution (solvent A: acetonitrile, solvent B: water added with 0.1% of formic acid; gradient: from 80% to 60% of acetonitrile in 7 min, return to the initial conditions in 2 min) at a flow rate of 1 mL min^−1^, detection performed at 254 nm.

As for RC-33 release studies, the IPs obtained by adding 176 mg of RC-33 to 10 mL of 1% *w*/*w* ALG and 17.6 mg of RC-33 to 10 mL of 1% *w*/*w* ALG were dialyzed towards a physiological solution (0.9% *w*/*v* NaCl in MilliQ water) for 96 h. At prefixed time periods (0.5, 1, 2, 3, 6, 24, and 96 h) an aliquot of the physiological solution was collected and drug concentration was quantified by the HPLC method above described. 

### 3.4. Preparation of the Polymer Solutions for Electrospinning

#### 3.4.1. Blank ALG/PEOs Solution

An aqueous solution of ALG/PEOs (BLANK) was prepared according to [44]. Briefly, ALG, h-PEO and l-PEO were dissolved in distilled water to achieve concentrations equal to 1%, 0.15%, and 1.98% *w*/*w*, respectively. P407 was added at the concentration of 2% *w*/*w* in order to reduce the surface tension of the ALG/PEOs blend. The solution was maintained under stirring overnight at room temperature before electrospinning.

#### 3.4.2. RC-33 Loaded ALG/PEOs Solutions

After the maximum binding capacity of ALG for RC-33 was identified, two RC-33 loaded ALG/PEOs solutions, containing 1% *w*/*w* ALG, 0.15% *w*/*w* h-PEO, and 1.98% *w*/*w* l-PEO, were prepared in distilled water by addition of RC-33 at two different concentrations. Such concentrations were calculated so that RC-33 could occupy 10% (10% LOADED) and 30% (30% LOADED) of ALG binding sites in a solution containing ALG at 1% *w*/*w*. Both LOADED solutions were maintained under stirring overnight at room temperature before electrospinning.

### 3.5. Characterization of the Polymer Solutions for Electrospinning

BLANK and LOADED solutions were characterized in terms of viscosity, surface tension, and conductivity.

#### 3.5.1. Rheological Analysis

Rheological analysis was performed by means of a rotational rheometer (MCR 102; Anton Paar, Turin, Italy) equipped with a cone plate combination (CP50-1, diameter = 50 mm; angle = 1°) as measuring system. Solution viscosity (η) was measured at 33 °C and increasing shear rates in the range 10–1000 s^−1^. Three replicates were performed for each solution.

#### 3.5.2. Dynamic Surface Tension Measurements

Dynamic surface tension measurements were carried out by means of an automatic tensiometer (DyneMaster DY-300; Kyowa Interface Science Co. Ltd., Saitama, Japan) at 33 °C. The analyses were performed by recording a surface tension value every 3 s up to 300 s. Three replicates were considered for each solution.

#### 3.5.3. Conductivity Measurements

Conductivity measurements were carried out by means of Mettler Toledo^TM^ FiveGo^TM^ F3 conductivity meter apparatus (Thermo Fisher Scientific). Three replicates were performed for each solution.

### 3.6. Fiber Preparation and Morphological Characterization

BLANK and LOADED fibers were prepared by using an electrospinning apparatus (STIKIT-40; Linari Engineering, Grosseto, Italy), equipped with a high-voltage power supply, a syringe pump, and a collector plate, covered by an aluminum foil. 

BLANK solution was electrospun at 20 cm (spinneret– collector distance) and 20 kV (applied voltage), while LOADED ones at 22 cm and 22 kV. All polymer solutions were pumped through a needle with a length = 15 mm and a gauge = 21 by setting the flow rate equal to 0.8 mL/h; the electrospinning process was performed at atmospheric pressure, maintaining constant temperature and relative humidity ranges (27–33 °C and 20–30%, respectively).

Morphological evaluation was performed by means of a scanning electron microscope (Tescan Mira3 XMU, Brno, Czech Republic). Fiber size was measured using the imaging analysis program ImageJ 2.0. Thirty fibers were considered for each sample.

### 3.7. Fiber Loading Capacity 

The loading capacity of 10% and 30% LOADED fibers was calculated after dissolution of known weighted LOADED fibers in a known volume of water. 

In details, the syringe, filled with the LOADED solution, was weighted before and after the electrospinning process in order to calculate the amount of the electrospun solution, and, thus, the theoretical amount of RC-33 into the resulting fibers; the latter were dissolved into Milli-Q water (fiber solution). An aqueous solution containing the theoretical concentration of RC-33 present in the fiber solution was prepared as reference (theoretical solution). 

The concentration of RC-33 in both fiber and theoretical solutions was identified through the HPLC method described in the Section 3.3. The % of RC-33 loaded into 10% and 30% LOADED before and after cross-linking was calculated according to the equation:

Loading capacity % = (*Af*/*At*) × 100 (1)

where *Af* was the area under the peak in the chromatographic profile of the fiber solution, while *At* was the area under the peak in the chromatographic profile of the theoretical solution. For each LOADED solution, three replicates were performed.

### 3.8. Assessment of Fiber Mechanical Properties

Fiber mechanical properties were assessed by means of a TA.XT plus Texture Analyzer (Stable Micro Systems, Godalming, United Kingdom), equipped with 5 kg load cells. Before testing, fiber thickness was measured by means of a Sicutool 3955G-50 (Milan, Italy) apparatus.

Each fiber was cut (1 × 3 cm) and then clamped on an A/TG tensile grips probe; an initial distance of 1 cm between the grips was set. The upper grip was raised at a constant speed of 10 mm/s. 

The maximum deformation force (Fmax) was measured and the deformation work was calculated as the area under the force vs. displacement curve (AUC); both tensile parameters were normalized for the fiber thickness. The percentage of elongation (Elongation%) was also determined according to the following equation [59]:

Elongation% = [Elongation at break − Initial length)/Initial length] × 100(2)

For each sample, three replicates were performed. 

### 3.9. Fiber Cross-Linking and Morphological Characterization

The cross-linking of BLANK and LOADED fibers was carried out according to a multistep protocol set up and described in [45]. Briefly, fibers were subjected to a pre-cross-linking treatment with absolute ethanol and, then, soaked in 2% *w*/*w* CaCl_2_ ethanol/water solutions. Finally, cross-linked fibers were washed with distilled water and dried at room temperature. The cross-linking of LOADED fibers was performed in the same cross-linking solutions mentioned above added with RC-33 at the same concentration present in the fiber. 

Known the solubility of RC-33 in ethanol and water, this expedient was necessary to avoid the complete release of RC-33 at the first step of soaking. 

Morphological analyses on cross-linked BLANK and LOADED fibers were performed (as described in the Section 3.6).

### 3.10. Film Preparation 

Films based on CS were prepared by casting method; two different *MW*s, low (CSL) and medium (CSM), were considered. Since CS is soluble at pH < 5.5, it was dissolved in an acetic acid (AA) or glutamic acid (GA) aqueous solution under gentle magnetic stirring at 70 °C overnight; glycerol was, then, added as plasticizer (Table 1). 

CS solutions were poured on flat glass plates (Petri dish, dimeter = 5 cm) and dried at room temperature until complete solvent evaporation. The volume of poured CS solution was calculated in order to guarantee the same amount of CS in all films prepared.

### 3.11. Dual-Functioning Scaffold Preparation

After cross-linking process (described in the Section 3.9), LOADED fibers, still wet, were soaked in the most promising CS solutions, when just poured into the glass plate. The scaffold thus produced was dried at room temperature until complete solvent evaporation. Morphological evaluation was performed as described in the Section 3.6.

### 3.12. Film and Dual-Functioning Scaffold Characterization

#### 3.12.1. Mechanical Properties

The mechanical properties of both the CS films and the dual-functioning scaffolds were assessed as described in the Section 3.8. Normalized Fmax, % elongation and normalized AUC were calculated. 

#### 3.12.2. Hydration Properties 

The dual-functioning scaffolds were subjected to hydration measurements at 37 °C by means of Franz diffusion cells (PermeGear, Bethlehem, Palestine). In particular, a weighted circular portion of the scaffold (=20 mm) was layered on a dialysis membrane in the apical chamber of a Franz cell. The receptor chamber was filled with phosphate buffer saline (PBS, pH 7.4), mimicking biological fluids and prepared according to European Pharmacopoeia 7.0. Scaffold portion was weighted after 1, 2, 3, 6, and 24 h contact with PBS solution. The PBS absorption was calculated, as % PBS uptaken using the equation:

% PBS uptaken = [(*Wf* − *Wi*)/*Wi*] × 100 (3)

where *Wf* was the weight of the scaffold portion after hydration, while *Wi* was the weight of the dried scaffold portion. For each sample, three replicates were performed. 

#### 3.12.3. Wettability

The wettability of both the CS films and the dual-functioning scaffolds was investigated by means of a Contact Angle Meter DMe-211 Plus (Kyowa Interface Science Co, Ltd, Niiza, Japan), according to the θ/2 method. The apparatus consists of a platform on which the scaffold was placed and a glass syringe, vertically positioned above the sample and able to dispense a defined volume of deionized water. A light source is positioned at one end of the platform, while the computer-interfaced camera is located on the other. 

Briefly, once the drop (10 μL) was released on the surface of the scaffold, a recording time of 20 s was set; instant images of the drop were automatically captured every second, starting from time 0, for the entire recording time. The FAMAS software allows to measure the contact angle (θ). Three measurements were performed for each sample.

#### 3.12.4. Biodegradation

The biodegradation of dual-functioning scaffolds was investigated after sample soaking in three different solvents: (i) PBS (phosphate buffer solution, without Ca^2+^ and Mg^2+^, Euroclone, Milan, Italy); (ii) H_2_O_2_ 1.25 mM in PBS; and (iii) β-*N*-Acetylglucosaminidase (from *Canavalia ensiformis* (Jack bean), NAGase, Sigma-Aldrich, USA) 5 U/L in PBS.

In details, weighted circular portions of each scaffold (0.32 cm^2^) were sterilized through UV irradiation for 24 h and, then, placed in a 96-well plate (Corning^®^ 96 Well TC-Treated MicroPlates, New York, NY, USA). The scaffolds were soaked in 200 μL of solvent (PBS, H_2_O_2_ or NAGase) and maintained in incubator (Shellab^®^ Sheldon^®^ Manufacturing Inc., Cornelius, OR, USA) (95% air and 5% CO_2_ atmosphere) at 37 °C for 7 and 14 days; in order to alter nor the pH neither the enzymatic unit, the solvent was changed every 24 h. After both 7 and 14 days, sample degradation was evaluated in terms of morphological alterations and weight loss. Each condition was investigated in triplicate. 

#### 3.12.5. In Vitro Cell Biocompatibility Assay

Cell biocompatibility has been assessed in human neuroblastoma SH-SY5Y cells. These cells were obtained from the European Collection of Cell Cultures (ECACC no. 94030304) and were cultured in a medium with equal amounts of Eagle’s minimum essential medium and Nutrient Mixture Ham’s F-12, supplemented with 10% heat-inactivated Foetal Bovine Serum (FBS), 2 mM glutamine, 0.1 mg/mL streptomycin, 100 IU·mL penicillin and non-essential amino acids at 37 °C in 5% CO_2_-containing and 95% air atmosphere. All culture media, supplements and FBS were purchased from Sigma Aldrich (Merck KGaA, Darmstadt, Germany). Cell viability was estimated by measuring LDH activity in the culture supernatants. The release of LDH by SH-SY5Y was measured by using a commercially available Cytotoxicity Assay Kit (Pierce, Thermo Scientific, UK), according to the manufacturer’s instructions. Briefly, a suspension of 200,000 cells/well was plated in a 24-well plate. By using a trans-well system, cells were exposed to CLS AA film/dual-functioning scaffold, previously sterilized through UV irradiation, for 24 h. After the exposure, a small amount of supernatant (50 μL) from treated and untreated wells was transferred to a new 96-well plate. An equivalent volume of LDH reaction mixture was added to each sample and the plate was incubated at room temperature for 30 min in the absence of light to allow color development. To block the reaction, 50 μL of stop solution, provided by the kit, was added to each condition. The amount of LDH within each supernatant was measured at 490 nm (680 nm background correction) using a Synergy HT microplate reader, BioTek (Winooski, VT, USA). To determine LDH activity, the 680 nm absorbance values (background signal from instrument) were subtracted from the 490 nm absorbance. The LDH released by untreated and treated cells was calculated and expressed as cell viability% vs. control.

### 3.13. Statistical Analysis

Whenever possible, experimental values of the various type of measures were subjected to statistical analysis, carried out by means of the statistical package Statgraphics 5.0 (Statistical Graphics Corporation, Rockville, MD, USA). In particular, one-way ANOVA–multiple range test was used. 

## 4. Conclusions

The formulation studies have led to the design of a dual-functioning scaffold characterized by both neuroprotective and neuroregenerative potential. While the loading of the S1R agonist should guarantee an attenuation of some secondary injury mechanisms exerting a neuroprotective activity, the scaffold architecture and surface properties and the presence of CS should be responsible for a neuroregenerative effect by supporting axonal outgrowth and sprouting. The research work focused on the formulation development of such system. 

ALG was chosen as the main fiber component for its capability to interact with the cationic RC-33: dialysis equilibrium studies demonstrated the formation of an IP between ALG and RC-33 and the capability of such product to control RC-33 release. The presence of drug at a concentration corresponding to 10% of ALG maximum binding capacity did not compromise the formation of homogeneous nanofibers by electrospinning and the possibility of cross-linking with calcium ions, making the fibers insoluble in physiological fluids.

Particular attention was devoted to the scaffold mechanical properties that play an important role during the system implantation. Among the CS-based films developed, those obtained from CS low MW (CSL) were characterized by a high flexibility/plasticity that is functional to an easy administration at the SCI site, avoiding the risk of film breaking during its handling. The nanofiber incorporation in CSL films was responsible for an increase in the scaffold mechanical resistance. 

Another important issue to be investigated is the scaffold residence time at the site of injury. The CS salification affected scaffold biodegradation rate: the scaffold based on CS salified with acetic acid (CSL AA) showed a slower degradation with respect to CSL solubilized in glutamic acid (GA). 

The developed scaffolds were characterized by topographical cues of nanometric magnitude, functional to axon outgrowth. Preliminary in vitro tests on neuroblastoma cell line pointed out that CSL AA based scaffold was biocompatible and characterized by cell viability values significantly higher than the positive control, represented by culture medium. Studies are ongoing to verify the scaffold capability to promote cell growth and to modulate the release of RC-33 in the simulated SCI environment.

## Figures and Tables

**Table 1:** Composition, expressed as % *w*/*w*, of CS-based solutions used for casting. AA e GA were used to solubilize CS.

	CS	Acid	Glycerol
CSL AA	2	1	1
CSL GA	1	0.7	0.7
CSM AA	2	1	1
CSM GA	1.4	0.7	0.7

## Supplementary Materials

The following are available online at https://www.mdpi.com/1660-3397/18/1/21/s1, Figures S1–S4.
