# Supplementary material for: Dual-Functioning Scaffolds for the Treatment of Spinal Cord Injury: Alginate Nanofibers Loaded with the Sigma 1 Receptor (S1R) Agonist RC-33 in Chitosan Films
Source: Mar Drugs. 2019 Dec 26;18(1):21. doi: 10.3390/md18010021 (PMC7024184; doi:10.3390/md18010021)
Supplement: Supplementary file 1 [file marinedrugs-18-00021-s001.pdf]

marine drugs

MDPI

Supplementary Data

Dual-functioning scaffolds for the treatment of spinal cord injury: alginate nanofibers loaded with the σ-1 agonist RC-33 in chitosan films

Barbara Vigani[1], Silvia Rossi[1*], Giuseppina Sandri[1], Maria Cristina Bonferoni[1], Marta Rui[1], Simona Collina[1], Francesca Fagiani[12], Cristina Lanni[1] and Franca Ferrari[1]

Synthesis of RC-33

As outlined in Figure 1S, the synthetic protocol provided a Heck reaction between the 4-bromobihenyl and the (E)-ethylcrotonate, followed by an *in situ* hydrogenation, using Pd/C 10% (w/w) as a catalyst. The saturated ester 1 underwent a base-mediated hydrolysis. Accordingly, the acid 2 was involved in an amidation reaction with the piperidine, in the presence of the TBTU, as a coupling reagent. Lastly, the reduction of 3 with $LiAlH_4$ was fruitful to achieve RC-33, which was converted into the corresponding hydrochloride salt. All the intermediates (1-3) and RC-33 were analyzed through HPLC, IR and NMR.

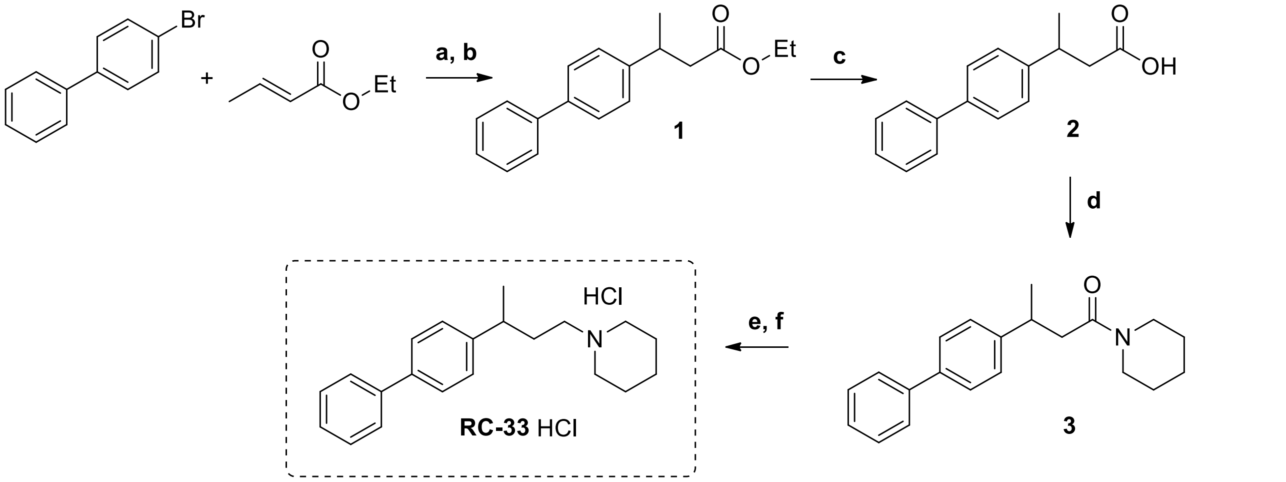

Figure 1S. Reaction conditions and reagents: a) TBAC, Pd(II)Acetate, NaAcetate, DMF, reflux; b) Pd/C (10% w/w); H2 atm.; abs EtOH; c) 3M NaOH, EtOH, rt; d) piperidine, TBTU, DIPEA, THF, 80 °C (mw); e) $LiAlH_4$, THF, rt; f) HCl 37%

In Figure 2S, the 1H NMR spectrum of RC-33 HCl (a) has been reported togheter with those of starting materials (b, c).

(a)

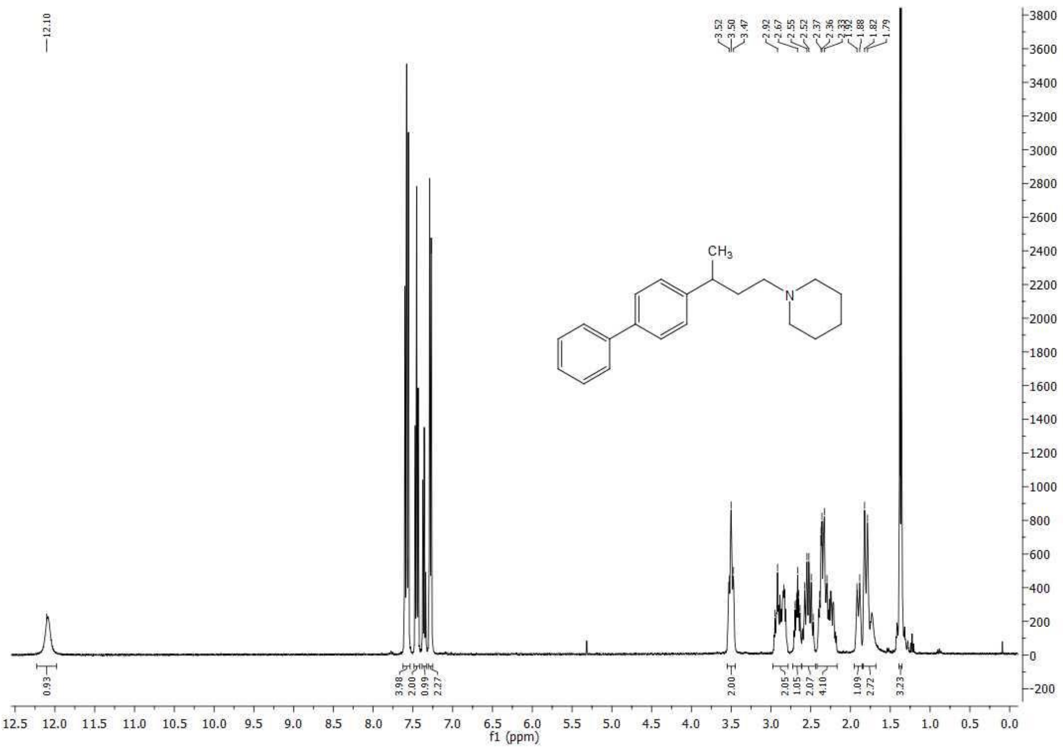

(b)

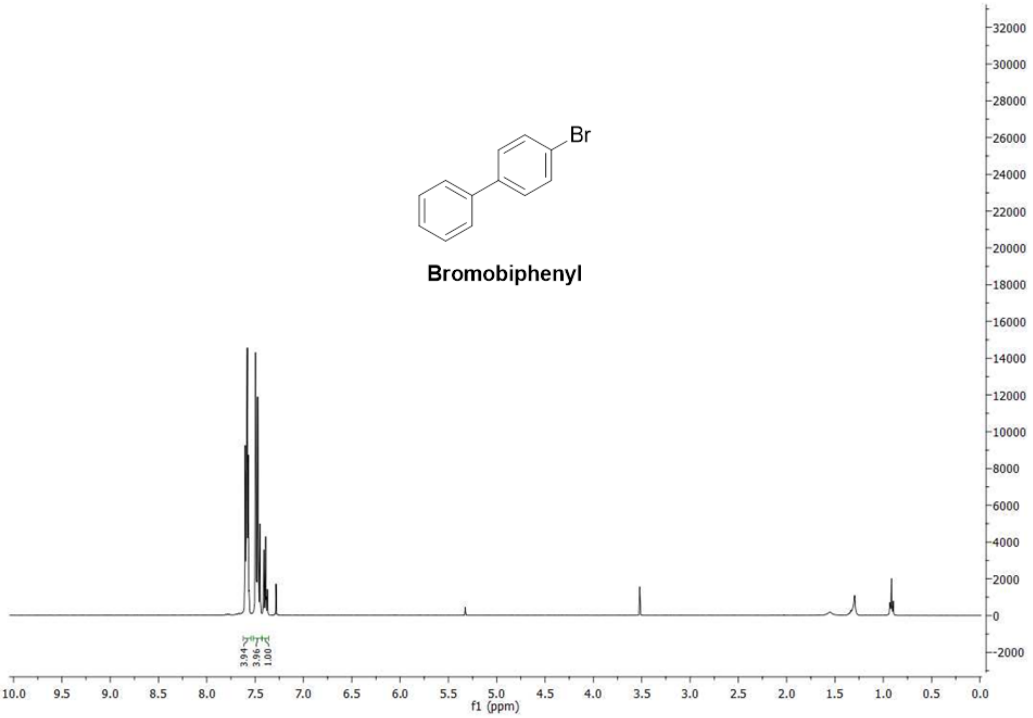

(c)

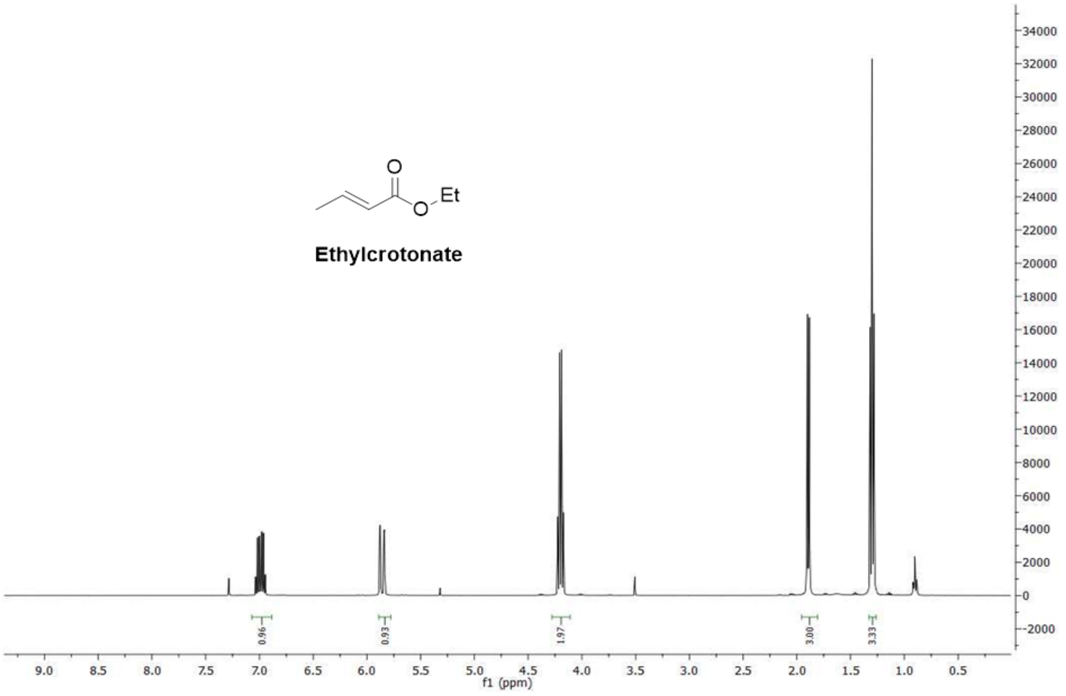

Figure 2S. 1H NMR spectrum of: (**a**) RC-33 HCl and (**b,c**) starting materials

CS solution rheological properties

The rheological behaviour of CS solutions used for film preparation was investigated by means of a rotational rheometer (MCR 102; Anton Paar, Turin, Italy) equipped with a cone plate combination (CP50-1, diameter =50 mm; angle =1°) as measuring system.

Sample viscosity (η) was measured at 25°C and increasing shear rates in the range 1–300 s^{-1}. Three replicates were performed for each solution.

Sample viscoelasticity was assessed by dynamic oscillatory measurements such as stress sweep test and oscillation test. In the stress sweep test, increasing stresses were applied at a constant frequency (1 Hz) and the elastic response of the sample, expressed as storage modulus G′, was measured. In the oscillation test, a shear stress, chosen in the linear viscoelastic region previously determined with the stress sweep test, was applied at increasing frequencies (1 to 20 Hz) and G′ (elastic storage modulus) and G″ (viscous loss modulus) profiles were recorded. Measurements were performed at 25°C in triplicate.

In Figure 3S and 4S the results obtained from the rheological characterization of the aqueous solutions of CS low (CSL) and medium (CSM) MW salified with acetic acid (AA) or glutamic acid (GA) are reported. As expected, an increase in polymer MW produces an increase in viscosity and viscoelastic properties, expressed by G′ and G″ values.

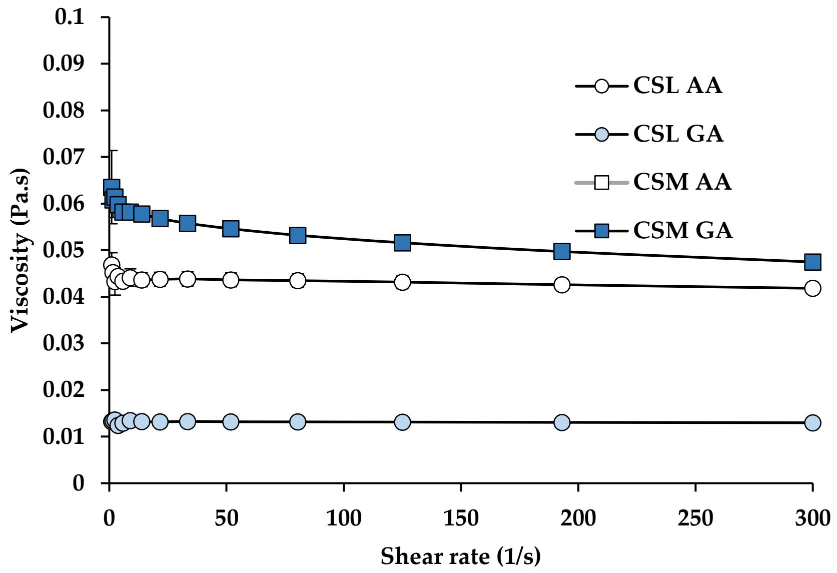

Figure 3S. Viscosity profiles of aqueous solutions of CS at two different molecular weights (CSL and CSM) salified with acetic acid (AA) and glutamic acid (GA) (mean values ± s.d.; n=3)

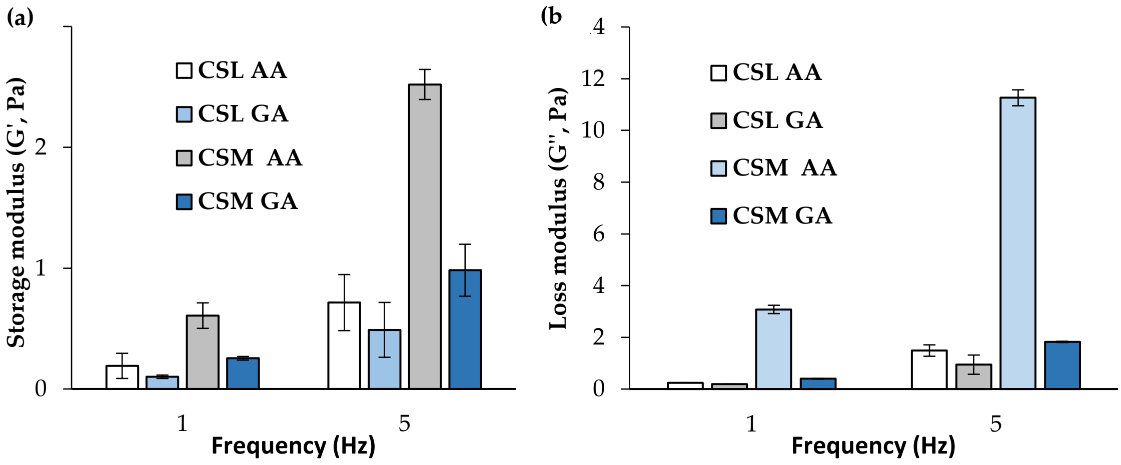

Figure 4S. Storage modulus (G′) and loss modulus (G″) values measured at 1 and 5 Hz frequency for all the CS solutions considered (mean values ± s.d.; n=3)
